# Simple re-instantiation of small databases using cloud computing

**DOI:** 10.1186/1471-2164-14-S5-S13

**Published:** 2013-10-16

**Authors:** Tin Wee Tan, Chao Xie, Mark De Silva, Kuan Siong Lim, C Pawan K Patro, Shen Jean Lim, Kunde Ramamoorthy Govindarajan, Joo Chuan Tong, Khar Heng Choo, Shoba Ranganathan, Asif M Khan

**Affiliations:** 1 Department of Biochemistry, National University of Singapore (NUS), Singapore; 2 Bioinformatics Centre, Life Science Institute, NUS, Singapore; 3 Computing Science Department, Institute of High Performance Computing, Singapore; 4 Department of Chemistry and Biomolecular Sciences, Macquarie University, Australia; 5 Department of Pharmacology and Molecular Sciences, Johns Hopkins University School of Medicine, USA; 6 Perdana University Graduate School of Medicine, Selangor, Malaysia

**Keywords:** Database archival, Re-instantiation, Cloud computing, BioSLAX, biodb100, MIABi

## Abstract

**Background:**

Small bioinformatics databases, unlike institutionally funded large databases, are vulnerable to discontinuation and many reported in publications are no longer accessible. This leads to irreproducible scientific work and redundant effort, impeding the pace of scientific progress.

**Results:**

We describe a Web-accessible system, available online at http://biodb100.apbionet.org, for archival and future on demand re-instantiation of small databases within minutes. Depositors can rebuild their databases by downloading a Linux live operating system (http://www.bioslax.com), preinstalled with bioinformatics and UNIX tools. The database and its dependencies can be compressed into an ".lzm" file for deposition. End-users can search for archived databases and activate them on dynamically re-instantiated BioSlax instances, run as virtual machines over the two popular full virtualization standard cloud-computing platforms, Xen Hypervisor or vSphere. The system is adaptable to increasing demand for disk storage or computational load and allows database developers to use the re-instantiated databases for integration and development of new databases.

**Conclusions:**

Herein, we demonstrate that a relatively inexpensive solution can be implemented for archival of bioinformatics databases and their rapid re-instantiation should the live databases disappear.

## Background

Other than the big well-funded institutionalized databases, few bioinformatics databases have longevity beyond several years. Small databases are particularly vulnerable. Valuable data and metadata become irretrievably lost leading to irreproducible scientific work and redundant effort [1-3]. This is unnecessary in view of the vast amount of affordable disk space and highly accessible cloud computing power in the market.

Recently, in 2009, the Asia Pacific Bioinformatics Network (APBioNet), Asia's oldest bioinformatics network and pioneer of the annual International Conference on Bioinformatics (InCoB) now in its twelfth year, initiated the effort for Minimum Information about a Bioinformatics Investigation (MIABi), building on earlier ideas [4]. The standards for transparency, provenance and scientific reproducibility amongst the bioinformatics and computational biology community were drafted and published a year later [5]. The MIABi standards were harmonised with the BioDBcore standards of the International Society for Biocuration (ISB) and BioSharing for use of standardized terminologies for infrastructural and informational interoperability [6].

In accordance to the MIABi standards, "when databases are described in a publication, a copy should be frozen, version-labelled and dated for reference", herein, we detail a simple methodology for archival and re-instantiation of small databases. Further, we provide a Web interface to demonstrate this functionality, with several exemplar databases to illustrate the utility of the system.

## Methods

**Figure 1:**
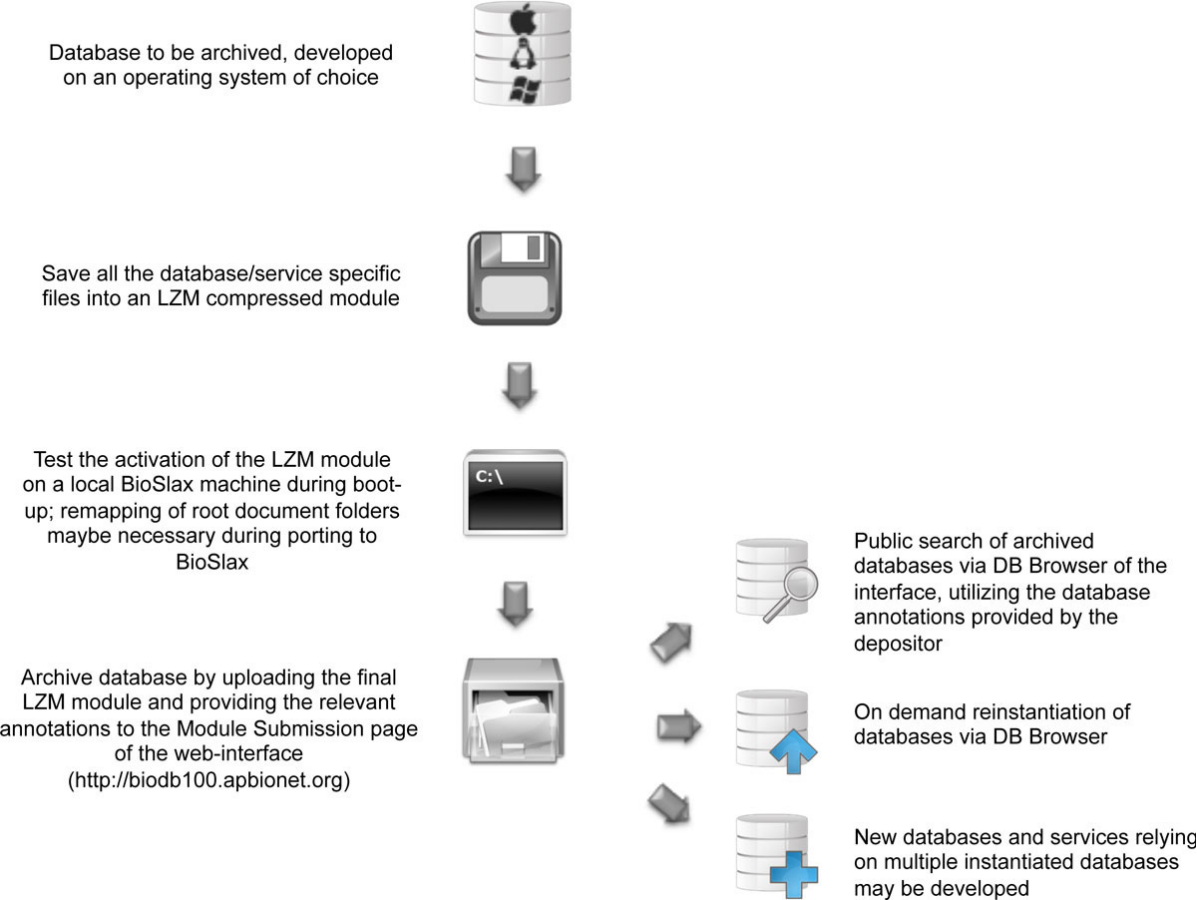
**A flowchart describing the utility of the re-instantiation system**.

Briefly, the re-instantiation system consists of a Web portal and a Cloud-based backend. The Web interface allows download of the base live operating system for database developers to build a compressed version of their database, upload their database, boot up a cloud instance, activate database and access the various functionalities (Figure 1). The content of the uploaded databases are vetted for extraneous files or programs that might be malicious before they are allowed for instantiation. Below we describe the different components of the re-instantiation system:

*(i) BioSlax*. The BioSlax 7.5 (http://www.bioslax.com) live operating system has been developed on a Slackware Linux base distribution called Slax (http://www.slax.org), to which we have added additional modules, including more than 200 bioinformatics software applications. BioSlax is freely downloadable and depositors of databases can choose to use it to develop their databases, or develop on another platform that can be ported to BioSlax. Upon boot-up, BioSlax un-packs, loads and activates all modules, including MySQL, Apache and Webmin servers. Other services can also be loaded and activated by adding additional BioSlax modules. Further, other operating systems will be explored in the future.

*(ii) Building databases as BioSlax LZM modules*. The BioSlax command "dir2lzm" is used to convert database specific files, other dependencies and other application services into LZM compressed file as BioSlax modules. Alternatively, database depositors can port pre-existing databases built on other platforms onto BioSlax, and save the difference as an LZM Slax module file that includes everything changed from the base LiveOS. The Slax "activate" command is used to unpack and uncompress all files and folders from the LZM file, and restart all relevant processes.

*(iii) Web interface*. A Web interface has been set up as the portal for users to access the re-instantiation system (http://biodb100.apbionet.org). Remote calls to the cloud server are made by public-private encryption key ssh secure login and remote execution of commands, called from CGI scripts written in bash and perl, with SSH, HTTP perl modules.

*(iv) A database of meta-information on uploaded databases and the compressed LZM files*. During the upload of the LZMs, meta-information about the submitters are obtained, such as their research group, author identifier (via http://aid.apbionet.org), and document/publication submission (via http://docid.apbionet.org). This meta-information is searchable and end-users can re-instantiate databases identified from the search, where the relevant LZM will be copied into a booted instance of a BioSlax OS and activated dynamically via the Web CGI call to the cloud server.

*(v) Xen Hypervisor or vSphere*. The cloud computing platform used is the popular open source full hardware virtualization software Citrix XenServer, based on Xen Hypervisor or VMware, based on vSphere, based on vSphere that provide the ability to create, deploy and manage the virtual machines on the cloud. For demonstration, we have set-up five instances of BioSlax virtual machines for remote booting on a private cloud that consists of 3 SuperMicro servers, each with dual Intel Xeon X5690 3.47 GHz 6-core processors (24 virtual processors through Intel Hyper-Threading technology), 145 GB of RAM and 1.8 TB of local storage. Xen's built-in "xe" commands are utilised in the Xenserver to poll halted BioSlax instances, activate on demand or shut down when idle. A Web interface for administrators to remotely start, stop or control virtual machine instances is provided (http://vmc.apbionet.org). We currently assign a public Internet Protocol (IP) address to each instance to enable external access to the databases.

## Results

We have successfully used our re-instantiable archival system for half a dozen extinct and extant databases (Table 1). Some databases were previously developed on non-BioSlax platforms, but were ported to BioSlax without difficulty. Hardcoded hyperlinks and directory paths were made relative in order for the unpackaged files to work properly. MySQL databases were compatible while other SQL databases required a SQL dump and recreation in MySQL format. Notably, none of the special SQL function calls not supported by MySQL were detected; otherwise, appropriate SQL rdbms would have to be installed into BioSlax as a separate LZM module when needed. Many databases are accompanied by search functions such as BLAST or other computational features, which were supported by BioSlax for the databases that we tested. Where this is not the case, the relevant programs can be compiled and added to the database LZM modules and activated accordingly. The database re-instantiation time was rapid, within 2 to 4 minutes. All database functionalities tested worked as per the original.

**Table 1:** Exemplar archived databases.

Exemplar Archived Databases	
**Type**	**Description**

Extant (available at published URL)	Allergen Atlas [8]
	Customary Medicinal Knowledgebase (CMKb) [9]
	MHC-Peptide Interaction Database-TR version 2 (MPID-T2) [10]
	Signal Peptide Database (SPDB) [11]
	STATdb: A specialised resource for STAT proteins (http://statdb.bic.nus.edu.sg/)
	Sub-Domain (http://chaos.bic.nus.edu.sg/domain/)
	Type III Secretion System Effector Database [12]
	NFκB Base (http://bioslax01.bic.nus.edu.sg/nfkb/)
Extinct (not available at published URL, but copy maintained elsewhere)	MHC-Peptide Interaction Database version T (MPID-T) [13]

List of extant and extinct databases successfully archived for rapid re-instantiation by use of our cloud computing prototype platform (http://biodb100.apbionet.org)

## Conclusions

Database longevity is a chronic problem within the bioinformatics community. In this report, we demonstrate that a relatively inexpensive solution can be implemented for archival of bioinformatics databases and their rapid re-instantiation should the live databases disappear. Organisations, such as the APBioNet, can maintain such databases on a low-cost system using cloud computing for a long period. APBioNet will use this re-instantiation platform for their future InCoB conference submissions, as part of its MIABi compliance, as well as part of its larger effort in the BioDB100 project [7], to build 100 MIABi-compliant interoperable databases.

## List of abbreviations used

(APBioNet): Asia Pacific Bioinformatics Network; (InCoB): International Conference on Bioinformatics; (MIABi): Minimum Information about a Bioinformatics Investigation; (ISB): International Society for Biocuration.

## Competing interests

TWT and AMK are honorary directors of Asia Pacific Bioinformatics Network Limited. The authors declare that they have no competing interests.

## Authors' contributions

TWT conceived the study, participated in its design and coordination. MDS and KSL implemented the prototype system. CX, CPKP, SJL, KRG, JCT, KHC, SR and AMK contributed one or more databases. AMK, MDS, KSL and TWT drafted the manuscript. All authors read and approved the final manuscript.

## Declaration

Publication of this article was funded by the EUAsiaGrid Project (FP7 of the EC) awarded to TWT.

This article has been published as part of *BMC Genomics *Volume 14 Supplement 5, 2013: Twelfth International Conference on Bioinformatics (InCoB2013): Computational biology. The full contents of the supplement are available online at http://www.biomedcentral.com/bmcgenomics/supplements/14/S5.
